# Hospital staffs’ perceptions of an electronic program to engage patients in nutrition care at the bedside: a qualitative study

**DOI:** 10.1186/s12911-017-0495-4

**Published:** 2017-07-11

**Authors:** Shelley Roberts, Andrea Marshall, Wendy Chaboyer

**Affiliations:** 1 National Centre of Research Excellence in Nursing, Menzies Health Institute Queensland, Griffith University, Gold Coast Campus, Gold Coast, QLD 4222 Australia; 2 Gold Coast Hospital and Health Service and School of Nursing and Midwifery, Griffith University, Gold Coast Campus, Gold Coast, QLD 4222 Australia

**Keywords:** Beside technology, Health information technology, Hospital staff, Nutrition care, Patient-centred care, Patient participation

## Abstract

**Background:**

Advancements in technology are enabling patients to participate in their health care through self-monitoring and self-management of diet, exercise and chronic disease. Technologies allowing patients to participate in hospital care are still emerging but show promise. Our team is developing a program by which hospitalised patients can participate in their nutrition care. This study explores hospital staffs’ perceptions of using this technology to engage patients in their care.

**Methods:**

This qualitative study involved semi-structured interviews with hospital staff providing routine nutrition care to patients (i.e. dietitians, nutrition assistants, nurses, doctors and foodservice staff) from five wards at a tertiary metropolitan teaching hospital in Australia. The hospital currently uses an electronic foodservice system (EFS) for patient meal ordering, accessed through personal screens at the bedside. Participants were shown the EFS program on an iPad and asked about their perceptions of the program, with questions from a semi-structured interview guide. Staff were interviewed individually or in small focus groups. Interviews lasted 15–30 min and were audio recorded and later transcribed. Data were analysed using thematic analysis.

**Results:**

Nineteen staff participated in interviews. Overall, they expressed positive views of the EFS program and wanted it to be implemented in practice. Their responses formed three themes, each with a number of subthemes: 1) Enacting patient participation in practice; 2) Optimising nutrition care; and 3) Considerations for implementing an EFS program in practice. Staff thought the program would improve various aspects of nutrition care and enable patient participation in care. Whilst they raised some concerns, they focused on overcoming barriers and facilitating implementation if the program were to be adopted into practice.

**Conclusions:**

Staff found an EFS program designed to engage patients in their nutrition care acceptable, as they saw benefits to using it for both patients and staff. Staff recognised characteristics of the program itself, as well as allocation of roles and responsibilities in operationalising it, were pivotal for successful implementation in practice. Their perspectives will inform program and intervention design, and implementation and evaluation strategies.

**Electronic supplementary material:**

The online version of this article (doi:10.1186/s12911-017-0495-4) contains supplementary material, which is available to authorized users.

## Background

In recent years there has been rapid advancement in health information technology (HIT), allowing patients to participate in their health care like never before [1]. Smartphone applications and wearable devices enable patients to self-monitor and set goals to improve health through diet, exercise and chronic disease management [2–4]. Some programs allow for information sharing between patients and practitioners, in order to provide tailored feedback and improve health care delivery [4, 5]. HIT interventions have been shown to positively impact on patient behaviour and improve patient engagement; but the majority of studies have been conducted among individuals living in the community [5]. Considering the success and eager uptake of HIT in this setting, it appears that hospitals and health care organisations are lagging behind [6]. Research on the use of HIT to engage hospitalised patients in their care is only emerging, but early work shows promise for using such technologies in the clinical setting [7].

In hospital, preliminary work has been done on the design, feasibility and acceptability of technologies to engage patients in areas of care such as surgical recovery [8, 9], falls prevention, discharge planning [10, 11], cardiovascular health [12–14] and medication safety [15]. A review of these studies indicated that using technology to enable active patient participation in their hospital care is both feasible and acceptable [7]. When patients participate in their care, they experience improved safety and less adverse events [16], better health outcomes and higher satisfaction with care [17]. Patient participation in nutrition care has also been shown to improve patients’ dietary intakes in hospital [18, 19]. This is a priority given inadequate dietary intake is the major risk factor for malnutrition, which affects 20–50% of hospitalised patients [20, 21] and increases morbidity, mortality, hospital length of stay and costs [22, 23]. However to our knowledge, no studies have yet used technology to engage hospitalised patients in their nutrition care.

Our team is developing a technology-based intervention that allows patients to participate in their nutrition care at the hospital bedside. The intervention is extends from our previous work indicating that patient participation in their nutrition care is feasible, acceptable, and likely to be effective in improving dietary intakes (citation masked for peer review). It is guided by an integrated knowledge translation approach [24]; informed by theories of self-efficacy [25] and HIT usability and acceptance; [26, 27] and underpinned by concepts of patient participation in care [28]. In line with these approaches, new technologies should undergo a series of iterative development and evaluation phases that involve end-user input to maximise usefulness and usability [29]. This study aimed to explore one group of end-users’, that is, hospital staffs’ perceptions of an intervention designed to engage patients in their nutrition care.

## Methods

### Study overview

This qualitative descriptive study [30] involved semi-structured interviews with hospital staff providing routine nutrition care to patients. The study received ethical approval from the participating health service (reference number HREC/16/QGC/118).

### Study setting

The study was conducted on five medical and surgical wards (medical vascular, oncology, orthopaedic, renal and respiratory) at a tertiary metropolitan teaching hospital in Australia. The hospital currently uses an electronic foodservice system (EFS) (Delegate Software Australia Pty Ltd) for patient meal ordering, accessed through patients’ Personal Entertainment System (PES) screens at the bedside. Patients have their own PES screen, which can be moved manually (via a repositionable arm) and accessed at any time. The EFS stores information such as foods and fluids ordered by patients at each meal and the nutritional content of each dietary item provided by the hospital. The only aspect patients currently use the EFS for is meal ordering.

### Intervention

Our team is developing a patient-centred program that allows patients to actively participate in their nutrition care by completing the Malnutrition Screening Tool (MST), dietary intake monitoring (intake tracking) and guided goal setting. Patients will complete these activities within the EFS program, accessed via patients’ bedside touch screens (PES screens). The MST comprises a few simple questions and generates a risk score, which will be available to clinicians for care planning and prioritisation. The intake tracking involves patients selecting the amount they have consumed (none, ¼, ½, ¾, all) for each dietary item, at each meal. The EFS calculates the total energy and protein consumed by patients from the data they have entered, which is available for both patients and clinicians to view, alongside the patients’ individual energy and protein requirements (calculated and entered into the EFS by the dietitian). The proportion of patients’ requirements met by their dietary intake will be presented as ‘nutrition goals’ and can be viewed at the patient interface (via the PES) for self-monitoring and to facilitate patient-centred discussions around nutrition during dietitian consults. Hence, the targeted end-users of this program are both patients and hospital staff involved in patient nutrition care. The program is being developed within the hospital’s existing EFS by expanding current and developing new functionalities. An iterative development and evaluation cycle is being used to design the program [26, 29] to incorporate end-users’ needs and maximise usability. A basic version of the program was developed for the purpose of end-user testing with staff (this study) and patients (reported elsewhere; citation masked). End-user feedback will be used to further develop and refine the program.

### Participants and recruitment

Participants included staff on study wards who were involved in patients’ nutrition care: dietitians, nutrition assistants, nurses, doctors and foodservice staff. With assistance from managers in each ward or department, purposive sampling was used to recruit participants from a mix of disciplines and staffing levels. Participants gave informed consent prior to participation. Recruitment stopped when data saturation was reached (i.e. data collection ceased when no new information was identified).

### Data collection

The Theoretical Domains Framework [31] underpinned the development of a semi-structured interview guide used for data collection (see Online Additional file 1). This validated framework is used to help explain human behaviour and is commonly used prospectively to identify what helps and hinders certain behaviours in behaviour change and implementation research [31, 32]. The guide included questions around three broad areas: nutrition care, patient participation and the EFS program. All interview data were collected by a trained interviewer with a background in dietetics. Data were collected at a time and place of convenience to staff, such as in a meeting room in the area where they worked. Staff were initially shown the proposed program on an iPad and the interviewer explained how each of the functions would be used to engage patients in their care. A conversational style of interviewing was used with the semi-structured interview guide and participants’ responses providing direction for the interviewer. Staff were interviewed individually (doctors, nurses, dietitians) or in pairs (nutrition assistants, foodservices). Interviews lasted 15–30 min and were audio recorded and later transcribed verbatim.

### Data analysis

Data were analysed using an inductive approach to thematic analysis [30, 33]. The lead author read and reread transcripts to become immersed in the data. Key quotes were highlighted and codes were developed based on verbatim statements of participants. Codes were grouped according to similarity into sub-themes then themes based on common threads throughout the data. Trustworthiness of findings was enhanced through frequent discussions among the research team to ensure codes, sub-themes and themes adequately described and encompassed data.

## Results

A total of 19 staff were interviewed, including ten registered nurses (seven bedside nurses and three in ward leadership roles), three dietitians (two junior and one senior), two nutrition assistants, two foodservice staff and two doctors (one intern, one resident). Most participants were female (*n* = 17, 89%). Dietitians, nutrition assistants and foodservice staff serviced a number of wards, whilst nurses and doctors worked on a single ward only. Nurses worked on the renal (*n* = 3), oncology (*n* = 2), medical vascular (*n* = 2), respiratory (*n* = 1) and orthopaedic wards (*n* = 2). The two doctors were from oncology and renal wards.

Overall, staff expressed positive views of the EFS program. They liked its various aspects, finding it easy to use and understand, and recognised benefits to using it for both patients and staff, all of which made it acceptable to them. Most staff had positive attitudes towards technology in general, acknowledging the move towards computerisation, particularly in hospitals. This general acceptance of technology extended to the EFS program, which was welcomed by staff as a ‘good idea’. Their responses formed three themes and various subthemes, outlined in Table 1.

**Table 1:** Themes and subthemes

Theme	Subthemes
1. Enacting patient participation in practice	a. Accepting and promoting patient participation in practice b. Adopting an individualised approach to participation c. The EFS program is a tool to enable patient participation in nutrition care d. Difficulty relinquishing control over information because of a lack of trust
2. Optimising nutrition care	a. Nutrition within the multidisciplinary team b. The EFS program can improve nutrition-related documentation c. The EFS program can improve information access and management to allow for more patient-centred nutrition care
3. Considerations for implementing the EFS program in practice	a. Helping patients overcome barriers to using the program b. Managing change in workloads and tasks c. Roles and responsibilities of staff in operationalising the program d. Requirements of the program

### Enacting patient participation in practice

The concept of patients participating in care was discussed often by staff, especially by nurses. Staff had positive views on active patient participation, expressing how they accepted and promoted it in everyday practice. They described how patients and families participated in care and explained the need for an individualised approach to participation. They spoke about how the EFS program could enable patients to participate in nutrition are. However, some staff found it difficult to trust patients and give them control over certain tasks.

#### Accepting and promoting patient participation in practice

Of all staff, nurses most frequently spoke about patient participation in care and expressed positive attitudes towards it, describing not only how they accepted it as part of routine care, but how they actively promoted it. Nurses strongly believed patients should be aware of and involved in their care. They spoke about building trust with patients and empowering and supporting them to participate. Dietitians also advocated participation and believed it empowered patients to contribute to decision-making and feel more in control of their care. Most staff recognised that participation benefited patients in terms of their health outcomes. Of the two doctors interviewed, one described having no experience with patient participation in care, whilst the other said it was important that patients were informed and interested in their care in order to improve outcomes. Nurses and dietitians most often spoke about how they enabled patients to participate in care; for example through self-monitoring, such as asking patients to keep their own fluid balance and food charts. Staff believed this made patients more aware of their condition and more compliant with their care. They said patients liked knowing their plan of care and in turn, they thought patients were more likely to participate meaningfully when they understood their condition and care plan.

*“…the existing food chart is filled by nurses and sometimes we get patients involved as part of helping them to become good self-managers, involved in completing their food chart.” (Dietitian 1).*

#### Adopting an individualised approach to participation

Nurses acknowledged that whilst patient participation was important, it depended on patients’ ability and motivation to participate. Some nurses thought it was the patient’s choice whether or not they would participate, whilst others described how they would deem a patient fit or not to participate depending on their condition. For example, some said they would not ask patients to actively participate when they were acutely unwell, as patients relied on nurses to get them through a ‘bad spell’. Some patients were seen to actively participate and ‘do it all for us’, which nurses encouraged. Whilst staff thought most patients (and their families) would like to contribute to care, they recognised that some preferred a passive role as they counted on staff, did not take an interest in their health care, or were not familiar with the concept of participation. Staff thought patients would be more likely to participate in an area of care they perceived to be important. They expressed that everyone participated differently due to different needs and abilities, so education needed to be individualised to each patient.

*“Initially they [patients] may not be up to it [participating] and that’s fine – they will waive their choice to.... then other days when they start to feel better…” (Nurse 7).*

#### The EFS program is a tool to enable patient participation in nutrition care

Staff explained how the EFS program could enable patient participation, which was seen to benefit patients and staff. They frequently spoke about it being able to give patients more control, ownership and responsibility for their care. They described how many patients were already enthusiastic about ordering their own meals via the EFS and that self-ordering was a way of participating by having independence and control over the nutrition they received. However, when patients received default meals as a result of not ordering via the system (i.e. they were unaware or unable) staff perceived this to be opposing patient-centred care. Self-completed MSTs and intake tracking were seen as acceptable ways to involve patients in their care and increase their awareness, understanding and perceived importance of nutrition. Patient-generated intake tracking and managing goals were seen as exceptionally beneficial. Staff thought many patients didn’t realise they weren’t eating enough, and self-monitoring could increase patients’ awareness of their nutrition needs and intake, which could motivate them to eat more. Staff also perceived functional benefits of the EFS that enabled participation, such as electronic meal ordering being visually and physically easier to see and complete than paper menus for older patients. Some staff thought the EFS may also be a source of entertainment, giving patients ‘something to do’ whilst in hospital.

*“I think it would be really, really beneficial because I know it would probably make patients more aware of their own care… It gives the ownership back to the patient a little bit, rather than the staff….driving things or doing a food chart.” (Nurse 9).*

#### Difficulty relinquishing control over information because of a lack of trust

Whilst most staff supported patients participating in their nutrition care using the program, some expressed they still wanted control over tasks such as MST completion and intake tracking. This mostly stemmed from concerns that data entered by patients may not be accurate. Some nurses had difficulty trusting patients and thought they still needed to help patients complete these tasks, or at least oversee or ‘check’ entered data before sending it on. Nurses thought poor ability to use the EFS, wishing to hide a nutritional problem from staff, or misinterpretation of questions could be potential reasons for patients providing inaccurate data. Dietitians and nutrition assistants also expressed some concern around the accuracy of patient-reported MST and intake data but overall still wanted patients to lead these tasks. Foodservice staff expressed very low trust in patients accurately reporting information.

*“I don’t think I would leave it [intake tracking] all up to the patients, because they can lie... if you want an accurate reading… you are better off leaving it to a staff member.” (Foodservice 1).*

### Optimising nutrition care

Staff explained how they strove to optimise nutrition care for their patients and how they thought the EFS program could help them achieve this. They spoke broadly about what nutrition care involved in their current practice, describing roles and responsibilities within the multidisciplinary team and attitudes towards nutrition. Overall, most staff expressed pride in the high quality of nutrition care they provided to patients and gave examples of this. Nurses were particularly prominent in nutrition care delivery and displayed a strong ownership of nutrition in practice. Staff also discussed how they thought the EFS program could be used to improve nutrition care by streamlining processes around documentation and management of information.

#### Nutrition within the multidisciplinary team

Staff described how they and others actively contributed to patient nutrition care as part of a multidisciplinary team. Dietitians were seen as the main providers of this care, responsible for overall nutritional management of patients. Nurses and nutrition assistants supported dietitians by collecting, documenting and communicating nutritional information. Nurses seemed central to many nutrition care processes such as risk screening, intake monitoring, nutritional assessment and education. Nurses strongly expressed an ownership of nutrition care, which was portrayed as more than a mere contribution to it. They described instances of critical thinking, clinical decision-making and active involvement in nutrition care that was self-driven, rather than just following dietitians’ orders. Staff highlighted that effective communication within the multidisciplinary team and good working relationships between nurses and dietitians were pivotal to creating a positive ward culture and providing optimal nutrition care. Staffs’ individual knowledge around nutrition seemed to impact on their attitudes towards it, which were subsequently seen by staff to affect ward culture. Most staff displayed high levels of nutrition knowledge specific to their area of care and acknowledged its importance for patients’ health and recovery; hence, nutrition was seen as a care priority for most. One doctor contrasted from the majority, stating nutrition was not a high priority and showing little interest or knowledge in the area. This doctor was dismissive of nutrition in practice and described ‘deferring the responsibility’ to someone else rather than being involved in aspects of nutrition care. Staff had varying opinions of the role of doctors; some thought they should play a role in nutrition care but currently didn’t, whilst others thought they contributed adequately. There was stark contrast in the two interviewed doctors’ approaches to nutrition care in practice.

*“I think each ward has a different culture, depending on the Nurse Unit Manager (NUM) and staff that are there. So, the renal ward is really good, they’ve got a great NUM and they’re a great team and they are all onto it – they help the patients on the PES and everything. Whereas other wards may not be nutrition focused… nutrition just gets forgotten.” (Nutrition Assistant 1).*

*“I like to know whether my patients are getting enough or dealing with appetite problems and I like to deal with that early, because we know that affects their healing and recovery and also pressure area care.” (Nurse 7).*

#### The EFS program can improve nutrition-related documentation

Staff, particularly nurses, described how they thought the program could help overcome existing barriers to nutrition-related documentation, mostly by reducing paperwork. They liked the electronic MST, describing it as a simple and useful tool that could save nurses time and work if patients completed it themselves. Staff frequently spoke about how the ‘intake tracking’ function could help overcome current barriers to completing paper food charts used for patient intake monitoring. Staff explained food charts were poorly completed as nurses were ‘too busy’; they lacked time and had high workloads, with large amounts of paperwork. The program was seen to potentially improve this issue, as some burden could be relieved by patients completing intake tracking. Staff thought patient-generated intake tracking and MSTs would be completed more often and more accurately than nurse-completed forms, for which completion and accuracy was considered poor, even by nurses. Nurses said they would prefer using the EFS over paper food charts to monitor intake (i.e. for patients unable to use the EFS) as it was quicker and easier. Whilst nurses applauded the idea of electronic intake monitoring, they didn’t want double-up of tasks; that is, they wanted the EFS to completely replace paper food charts.

*“It would be a lot easier to look at that [intake tracking] than a food chart. Also, obviously nurses are very busy, so if the patient is just doing it themselves then that might be a better prospect.” (Doctor 2).*

#### The EFS program can improve information access and management to allow for more patient-centred nutrition care

Improved access to and management of nutritional information was another benefit staff saw the program having on the care they provided to patients. In particular, the ability for nurses, doctors, dietitians and other health professionals to access patients’ dietary intakes from any computer via the intake tracking function was seen as a major benefit and was preferred over paper food charts. Staff thought this would allow for earlier identification of patients eating poorly and better care provision. They also thought the ability to view nutritional information of foods on the hospital menu was beneficial to both patients and staff. Dietitians were very interested in the program and thought it could streamline nutrition assessment, allow for individually tailored nutrition care and education, and increase patient participation in care; particularly the goal-setting function. Staff thought the program provided patients with more information about their care. For example, nurses liked the brief explanation of why it was important for patients to answer MST questions on the EFS, which nurses didn’t have time to explain when completing on paper. Nurses wanted the ability to see whether patients had completed their electronic MST or not, so they could provide assistance or complete it for them if necessary. Staff were concerned with the management of electronic data; they wanted information to ‘go somewhere’ and stressed the importance of data being purposefully used so it was not forgotten or ‘lost’ in cyberspace. For example, staff wanted MST scores of all patients to be visible in a central location and automatically generate dietitian referrals for patients at risk. They also wanted intake tracking information to be available to all staff after patients had entered it.

*“If it [MST] was electronic I would expect it would go automatically through to the dietitian… if it was missed by a dietitian because it was the weekend, then the nurse could pick that up.” (Nurse 8).*

### Considerations for implementing the EFS program in practice

Staff spoke about issues they saw as important to consider in the effective implementation of the EFS program in practice. Firstly, they described potential barriers that may be faced by patients in using the program and how these may be overcome. They expressed their opinions of who should be responsible for various new tasks and roles the implementation of the program may introduce, and how changes in workload may be managed. They also explained the requirements of the program needed for it to be effective and accepted.

#### Helping patients overcome barriers to using the program

Staff perceived potential barriers to patients using the EFS to participate in care; however, they focused on strategies to overcome these. Patients’ ability to use the technology was frequently mentioned as a potential barrier. Older age; unfamiliarity with technology; being acutely unwell, cognitively impaired, frail or dependent; and physical limitations such as poor eyesight or inability to access the touch screen were seen to impact on patients’ ability to use the program. It was also noted by staff that patients with these issues were likely to be at higher risk of malnutrition. Staff suggested that having a staff member or relative assist patients with these tasks could help overcome barriers. Nurses highlighted that even older patients could still be taught how to use the system and gave examples of how patients had learned and become familiar with the EFS during their admissions. Some nurses thought poor compliance may be a barrier to using the program. They specifically mentioned unmotivated chronic disease patients for whom they regularly cared for on their wards, and whom did not follow health care professionals’ (HCP) advice. A poor attitude or low care factor about their health was seen to contribute to this. Whilst some staff thought patients could be encouraged or motivated by the goal-setting component of the program, others believed that there was no hope of changing these patients’ attitudes.

*“I would say there definitely would be a percentage [of patients] that would find it difficult to fill them in. But I think the nursing staff could certainly assist in those cases if we are already filling in a food chart anyway.” (Nurse 9).*

#### Managing change in workloads and tasks

Staff perceived a number of new tasks and changes in workload that could arise with the implementation of the EFS program. Many staff welcomed changes of tasks such as the MST and intake monitoring from paper-based to electronic as they thought it could streamline care and reduce their workloads. Any potential additional work was seen to be negated by the time potentially saved with the program’s introduction. Staff also believed any extra work would be worthwhile if it meant improving patients’ nutrition care. The main new task described was assisting patients who were unable to use the EFS themselves. However this was not perceived as a burden to most staff; it was seen as being quicker and easier alternative to paper MSTs and food charts. Staff thought electronic MSTs would increase the number of patients screened for malnutrition, with most patients completing their own MST and nurses assisting in other cases. Whilst this was seen as a positive, nutrition assistants and dietitians were somewhat concerned that increased screening rates would increase their workloads. However, patient care was still the priority, and these staff acknowledged the program could save them time in other areas, such as intake tracking (as nutrition assistants performed meal audits on patients) and screening, referrals and prioritising patients (for dietitians).

*“Part of our nutritional screening is asking those two questions… so if that was introduced as opposed to what we do now, I don’t think that would take more time. In fact I think it would probably save time really, if the patient is doing it.” (Nurse 10).*

#### Roles and responsibilities of staff in operationalising the program reflects current nutrition practice

As staff saw changes in workloads and emergence of new tasks with the introduction of the EFS program, they subsequently gave their perspectives of whose role it should be to perform these. Dietitians were seen as the main users of the EFS and hence, the overall managers of its operation and use. Most staff thought as dietitians would be requesting and using dietary information, it was their responsibility to allocate patients to intake tracking and train them on how use the EFS. However, some nurses thought this would be their role as they already orientated patients to the PES on admission. Several staff spoke about a shared responsibility between nurses, dietitians and nutrition assistants in training patients. Overwhelmingly, nurses took on the responsibility of various roles in supporting patients to use the EFS and managing information, consistent with their approach to nutrition care in general. Most staff agreed that as MSTs and food charts were currently nurses’ roles, they should continue to be responsible for these tasks after introduction of the program. Nurses said they would provide follow up, support and assistance to patients in continued use of the EFS and ensure tasks (MSTs and intake tracking) were completed. Nurses explained that for patients who were unable to participate they would take over their care anyway; for example, they already completed patients’ food charts so completing intake tracking was not much different. Many staff also thought there was a role for family members in using the EFS. They told of how families already helped patients with electronic meal ordering and suggested they could also be involved in intake tracking and MST completion via the EFS. There was confusion around foodservice staffs’ role in assisting patients with tasks such as electronic meal ordering; as one foodservice staff member stated, “Are we even allowed to help patients?”. Other staff thought foodservices could play this role as they used to help patients complete paper menus. However, foodservice staff explained since electronic meal ordering was introduced, they no longer helped patients as staff themselves didn’t know how to use the EFS or thought it was nurses’ responsibility.

*“I think we can assist them. But initially, the first person to bring that out, whether the dietitian wants it, needs to be the educator.” (Nurse 2).*

#### Requirements of the program

Staff perceived a number of qualities the program should possess in order to make it useful and usable for patients, families and staff. Simplicity was seen as a major facilitator to patients being able to learn how to use the system. For example, staff thought the MST function was particularly simple and most patients would be able to complete it. Staff thought the program should be ‘user friendly’ and easy to understand and navigate. Staff also proposed functionalities specific to their area of care; for example, renal nurses wanted the ability to track patients’ fluid intake. Others suggested pop-up reminders for patients to complete tasks such as the MST and intake tracking and tailored tips for patients on how to improve their nutrition. Dietitians thought it would be useful to highlight high energy / high protein foods on the electronic menu to help patients with their dietary choices.

*“We would have to make this software program quite basic so people that aren’t that familiar with technology find them easy to navigate.” (Nurse 9).*

## Discussion

This study explored hospital staffs’ perceptions of using an EFS program to engage patients in their nutrition care. Three themes emerged from interview data: 1) Enacting patient participation in practice; 2) Optimising nutrition care; and 3) Considerations for implementing an EFS program in practice. Staff generally expressed positive views of the program and spoke about how it could contribute to better nutrition care and enable patient participation. Most staff described a hands-on approach to nutrition care, especially nurses. Staff promoted patient participation in their practice and liked the idea of using the EFS to involve patients in care. Importantly, staff discussed a number of considerations for implementing the program in practice; perspectives that will be invaluable to the design of the program itself and its implementation and evaluation strategies. These findings will also be useful for others looking to develop and implement patient participatory HIT in the hospital setting.

### Technology for patient participation in care

The patient-centred EFS program evaluated in this study has a strong focus on engaging patients in their own care, which is becoming inherent in many HIT programs now and into the future. As such, the program was seen by staff to facilitate participation in several ways, many of which aligned with the dimensions of patient participation as identified in a concept analysis [28]. Staff thought the program could improve information access and management, for both patients and for staff providing nutrition care. Information sharing, that is, a meaningful exchange of information and knowledge between patient and HCP is one of the four dimensions of patient participation [28]. The EFS program was seen to improve patients’ knowledge and awareness of nutrition by providing access to information such as their personal nutritional needs, intake and dietary options. For staff, access to patient-specific information could help plan care and was a medium for education. A realist review of studies using technology to engage hospitalised patients in their care found that technology-based interventions employed this strategy (i.e. information and knowledge exchange) through information sharing, assessment and feedback, and tailored education [7].

Another dimension of participation is active mutual engagement in intellectual and/or physical activities [28]. In this study, dietitians spoke about engaging patients in intellectual activities such as educating them about their nutritional needs vs. intake (goal setting) and nurses spoke about physically helping patients use the EFS to engage in the program. Patients often require the support of staff when engaging with technology-based interventions, and they wish to maintain relationships with HCPs in using them [12]. That is, patients do not want technology to replace HCPs, but prefer it as a tool to support staff in providing care [7].

A third dimension, that is surrendering of some power or control by HCPs is required to enable participation [28]. Interestingly in this study, some staff were less willing to surrender control of certain tasks (MST and intake tracking) than others. Staff spoke about giving patients responsibilities in their current practice (such as keeping their own fluid balance or food charts), but some were hesitant to pledge control to patients over tasks within a system that was not yet implemented.

Finally, a trusting, mutual and respectful relationship between patient and HCP an important dimension of participation [28]. In this study staff spoke about knowing their patient, building trust, and empowering and supporting them to participate. They discussed how patients relied on them when they were unable to participate, and how understanding their patient allowed them to tailor activities to accommodate for differing abilities to participate.

### Technology-based decision aids

The EFS program could also be seen as a decision aid for both patients and HCPs, to promote participation. That is, it is a tool that can be used to facilitate informed and shared decision-making between patients and practitioners [34]. The information provided by the EFS program can be used by HCPs to plan care and enable patients to understand more about their nutrition whilst in hospital. The proposed goal-setting function can be used by patients and HCPs together, to provide information and options about potential avenues for nutrition care. Studies have found that decision aid systems providing advice for both patients and HCPs are more likely to be successful [35].

A Cochrane review found that compared with standard care, decision aids resulted in improved knowledge, more accurate risk perceptions and lower decisional conflict among patients; more patients choosing options aligning with their values; and less patients being passive in decision making [36]. These benefits are reflected in staff perceptions of the EFS program in the current study. Staff believed the program may improve patients’ knowledge, awareness and perceived importance about nutrition, which could help them make decisions about what to order or eat. This was seen to allow patients to be more active and in control of the nutrition they received, in order to improve health outcomes.

### Uptake of new technologies

Staff were generally very accepting of the EFS as a means of involving patients in their care, and welcomed its adoption for various reasons. Uptake of new innovations is complex, and several theories are used to understand how and why new technologies are (or are not) adopted in practice. According to Rogers, the uptake of new innovations can depend on characteristics of the innovation, individual adopters and the organisation [37]. In this study, staff perspectives were particularly focused on characteristics of the EFS program.

Rogers suggests relative advantage, low complexity, compatibility, observability and trialability of new innovations are likely to influence their adoption [37]. When discussing how the program could be implemented in practice, staff in this study highlighted the importance of it being easy to use (low complexity/ease of use) and linking in with existing electronic systems in the hospital (compatibility). They spoke about being able to use a tailored, individualised approach when engaging patients with the EFS, as ‘every patient is different’ (trialability). Finally, staff thought they would be able to see the impact the program had on their nutrition care practices through the benefits outlined above (observability). An interpretive review of 13 systematic reviews of issues surrounding HIT implementation in health organisations also found that the majority of end-users are accepting of technology, and successful uptake depended on characteristics of the technology itself, social aspects and organisational factors [38]. Characteristics of technologies were consistent with the current study and included usefulness and relative advantage over existing practices, ease of use, compatibility with existing systems and processes, demonstrable benefits and adaptability [38].

Similarly, the Technology Acceptance Model postulates perceived usefulness and ease of use affect adoption of new technologies [39]. A systematic review of factors influencing HCPs’ adoption of HIT concluded perceived usefulness/benefits was the most common facilitating factor, followed by perceived ease of use [40]. Others reporting staff perceptions of clinical information systems found perceived ease of use and usefulness impacted on staff attitudes, satisfaction and behavioural intention towards systems [41, 42]. In our study, staff found the EFS program acceptable when they saw benefits to using it; for example, they thought it could reduce time and paperwork, improve information access and management, and enable patient participation in care. That is, they perceived the program to be superior to current nutrition-related practices (relative advantage/usefulness).

Staff frequently spoke about how they thought the EFS program could be implemented into routine practice. Their perspectives aligned with Normalisation Process Theory, which is used to understand how new technologies are integrated into systems to become usual or ‘normal’ practice [43]. The theory consists of four main constructs (coherence, cognitive participation, collective action, reflexive monitoring), each with a number of components. Interestingly, staff were fairly optimistic about the program and focused on overcoming barriers and facilitating implementation. Staff made sense of the program (coherence) by considering how using it would differ from current practice (differentiation); by describing their own specific tasks, roles and responsibilities in operationalising it (individual specification) and how the multidisciplinary team should work together to achieve success (communal specification); and by recognising the value, benefits and importance of the program to patients and staff providing nutrition care (internalisation). Nurses were forward in accepting responsibility for a number of tasks and saw many benefits to using the EFS program in their practice. Throughout interviews, staff displayed cognitive participation in the program; they ‘bought into it’ as they thought it was acceptable (enrolment), discussed responsibilities for leading/driving it (initiation), expressed how they would contribute personally (legitimation) and described the actions needed to sustain its use in practice (activation). Staff also gave in-depth explanations of the operational work needed to enact practices relating to its implementation (collective action). They spoke about the interactional work between staff, the EFS and nutritional practices that would be needed to operationalise the system in routine care, as well as allocation of tasks and resources. Reflexive monitoring was not applicable as the program was not yet implemented. Other studies have also found constructs of Normalisation Process Theory explain why nutritional [44] or technology-based [45] innovations were (or were not) successfully adopted into routine practice.

Interestingly, some of the concerns and benefits of the EFS program staff perceived in the current study are comparable to previous research [46, 47]. A systematic review of HCPs’ perceptions of engaging patients in care using electronic portals found that in prospective studies (i.e. when portals had not yet been implemented), HCPs were concerned with the accuracy of patient-entered data, the potential increase in workload, and the liability and roles around tracking and acting on clinical information in the system [46]. However, the review found that in retrospective studies, these concerns were not justified and in fact portals were perceived by patients and staff to be very useful [46]. In our study, staff raised the same concerns. However, despite this being a prospective study exploring staffs’ perceptions of a program not yet implemented, staff were overwhelmingly accepting of the program. Any issues or barriers were outweighed by perceived benefits and were seen as manageable, with staff suggesting ways to facilitate the program’s implementation and use. The benefits of the EFS program staff perceived were comparable with another study on staffs’ perceptions on the use of electronic dietary assessment tools in primary care [47]. In that study, staff thought patient-generated dietary intake monitoring would increase patients’ awareness of what they were eating and motivate them to improve their dietary habits [47], consistent with staff perceptions in our study. Staff in both studies also perceived electronic dietary assessment tools would be more efficient, would improve the quality and quantity of dietary information available to HCPs, and would enable them to provide individually tailored education [47].

### Limitations

While there are a number of strengths to this study such as sampling a wide variety of hospital staff and undertaking a rigorous analytic process, it has several limitations. This was a relatively small study of 19 staff from one hospital in Queensland, Australia on their views of an EFS program. Whilst staffs’ perceptions are specific to this particular EFS program, which is not currently widely available, the findings may be important for the future (when it does become available) and may have applicability for the use of technology-based interventions to engage patients in care more broadly. It is possible that some views were not represented in our sample, however we used purposive sampling to improve generalisability and continued data collection until saturation was reached, which may have increased the relevance of our findings for other similar settings. Finally, nurses’ views emerged most strongly from the data, which may reflect the larger sample of nurses (*n* = 10), but may also be due to the active approach they expressed in providing nutrition care.

## Conclusions

This study explored the perspectives of hospital staff on using an EFS program to engage patients in their nutrition care. Overall staff found the program acceptable as they saw benefits to using it (for both patients and staff) in terms of improving nutrition care-related tasks and facilitating patient participation. Staff expressed that certain characteristics of the program itself, as well as the allocation of roles and responsibilities in operationalising it, were pivotal for its successful implementation and sustainment in practice. Staffs’ perspectives around implementing the program, particularly around its requirements, enabling patients to use it, and managing new tasks, roles and responsibilities will inform not only the design of the EFS program and the overall intervention it will be part of, but also its implementation and evaluation strategies.

## Additional file

Additional file 1: Semi-structured interview guide. (DOCX 21 kb)

## Abbreviations

EFS: Electronic foodservice system
HCP: Health care professional
HIT: Health information technology
MST: Malnutrition screening tool
PES: Personal entertainment system
