# Supplementary material for: Hospital staffs’ perceptions of an electronic program to engage patients in nutrition care at the bedside: a qualitative study
Source: BMC Med Inform Decis Mak. 2017 Jul 11;17:105. doi: 10.1186/s12911-017-0495-4 (PMC5504779; doi:10.1186/s12911-017-0495-4)
Supplement: Additional file 1: — Semi-structured interview guide. (DOCX 21 kb) [file 12911_2017_495_MOESM1_ESM.docx]

**Additional file 1: Semi-structured interview guide**

Interviews were conversational in nature, with the semi-structured guide providing some direction for the interviewer when conversation on each topic ended. The interviewer started by asking broad questions and allowing the participant to speak about each topic before asking more direct questions from the table below. As each interview was different, the following represents an example of questions that may have been asked of participants (i.e. not all questions were asked of each participant).

*Prelude:* The focus of this interview is your perspectives of patients using the hospital’s electronic foodservice system to enter their food intake information and complete their own Malnutrition Screening Tool (MST). We are interested in your opinions; there are no right or wrong answers. What you tell me will remain confidential and if used in any report will be anonymous.

*Broad questions to start interview:*

- Can you tell me what you know about patient-centred care / patient participation in care?
- How important do you think it is for patients to participate in their nutrition care?
- What are your thoughts on the program you just saw, in relation to engaging patients in their own nutrition care?

Table 1: Direct questions around nutrition care, patient participation in care and the EFS, based on the Theoretical Domains Framework

| **Domain** | **Core Questions** |
| --- | --- |
| Knowledge | What do you know about patient participation in care?  Why do you think patients need their food intakes monitored in hospital?  Why do you think malnutrition screening is conducted in hospitals? |
| Motivation and goals | How important do you think it is for patients to participate in their own care?  What do you perceive to be the importance of food intake monitoring for patients?  What do you perceive to be the importance of malnutrition screening? |
| Professional role and identity | What is your current role in helping patients use the EFS (if any)?  Who familiarises patients with the EFS (i.e. for ordering meals) or helps them if they encounter problems?  Do you see any role overlap or gaps between yours and other professions in helping patients use this system?  What is your current role (if any) in monitoring and recording patient intakes? In conducting malnutrition screening?  What professions do you think food intake monitoring/malnutrition screening are most important for?  What do you think your role would be if this patient-recorded intake function was rolled out for patients requiring intake monitoring?  Do you think this technology would make your job easier or harder? Why? |
| Emotion | Does anyone you work with have particularly strong feelings about patient intake monitoring (either positive or negative)?  Does anyone you work with have particularly strong feelings about malnutrition screening? |
| Social influences | What do your peers think about food intake monitoring, and how is this enacted on the ward?  Is there anyone on the ward whose views or behaviours influence how you think about food intake monitoring?  What do your peers think about the electronic meal ordering system?  Is there anyone on the ward whose views or behaviours influence how you think about electronic meal ordering?  What is the general feeling on the ward regarding malnutrition screening? How is this enacted on the ward?  Is there anyone on the ward who influences whether MSTs are completed or not? |
| Behavioural regulation | Are there things that make it easier or more difficult to keep food charts / complete MSTs on the ward?  Are there any ward guidelines, policies or training about keeping food charts / completing MSTs?  Are there any procedures or guidelines for familiarising patients with the EFS (i.e. for meal ordering) on the ward? |
| Beliefs about consequences | Do you think there are any benefits of food intake monitoring for patients?  If a dietitian has asked a food chart to be kept for a patient and it isn’t completed, do you think there are any negative consequences to that? What are they?  If a patient isn’t shown how to order their meals electronically, are there any consequences to this?  If MST is not completed, are there any consequences to this? |
| Skills | Do you feel confident in your ability to keep a food chart / monitor food intake / conduct MST?  Do you think members of your profession have these skills?  What do you do if you are not sure what the patient has eaten?  Do you think patients possess the skills to monitor their own intake / complete their own MST?  Do you think patients possess the skills to order their meals electronically? |
| Environmental context and resources | What resources are required to monitor a patient’s intake currently?  Is there anything about your ward (or workplace) in particular that makes this task easy or difficult?  Is there anything that makes malnutrition screening easy or difficult?  Do you find the electronic foodservice system to be a help or a hindrance? |
| Beliefs about capabilities | Do you feel that you are able to provide patients with adequate training to order their meals electronically?  Do you feel you could surrender the responsibility of completing MST to patients, for them to complete? |
| Memory, attention, decision processes | How important is training patients to use their electronic foodservice system to order meals, compared to other duties around admission time?  Are there any tasks that get in the way of showing patients how to use their PES?  Are there things that get in the way of doing MSTs / keeping food charts? |

**Example prompts:** Can you tell me a bit more about this? What do you mean by……? How/why?
